# Analysis Comparison for Rapid Identification of Pathogenic Virus from Infected Tissue Samples

**DOI:** 10.3390/diagnostics12010196

**Published:** 2022-01-14

**Authors:** Junji Hosokawa-Muto, Yukiko Sassa-O’Brien, Yoshihito Fujinami, Hiroaki Nakahara

**Affiliations:** 1 Fifth Biology Section, First Department of Forensic Science, National Research Institute of Police Science, Kashiwa 277-0882, Chiba, Japan; fujinami@nrips.go.jp (Y.F.); senju@nrips.go.jp (H.N.); 2 Laboratory of Veterinary Infectious Disease, Tokyo University of Agriculture and Technology, Fuchu 183-8509, Tokyo, Japan; sassa_y@cc.tuat.ac.jp

**Keywords:** tissue sample, infectious disease, forensic, virus, quantitative PCR, exhaustive gene amplification, next-generation sequencing, read, detection, identification

## Abstract

When examining infectious samples, rapid identification of the pathogenic agent is required for diagnosis and treatment or for investigating the cause of death. In our previous study, we applied exhaustive amplification using non-specific primers (the rapid determination system of viral genome sequences, the RDV method) to identify the causative virus via swab samples from a cat with a suspected viral infection. The purpose of the current study is to investigate suitable methods for the rapid identification of causative pathogens from infected tissue samples. First, the influenza virus was inoculated into mice to prepare infected tissue samples. RNA extracted from the mouse lung homogenates was transcribed into cDNA and then analyzed using the RDV method and next-generation sequencing, using MiSeq and MinION sequencers. The RDV method was unable to detect the influenza virus in the infected tissue samples. However, influenza virus reads were detected using next-generation sequencing. Comparing MiSeq and MinION, the time required for library and sequence preparation was shorter for MinION sequencing than for MiSeq sequencing. We conclude that when a causative virus needs to be rapidly identified from an infectious sample, MinION sequencing is currently the method of choice.

## 1. Introduction

When identifying a pathogenic virus from an infectious disease sample, the virus species is first estimated from the clinical course and the presenting symptoms of the patient, various tests and analyses of the pathological findings from the sample, and finally, the application of methods such as immunochromatography, ELISA using a species-specific antibody, and PCR using species-specific primers [1]. In the case of bacterial agents, universal genes, such as the 16S ribosomal RNA gene and other genes common to bacteria, can be used for species estimation, but there are no such universal genes among viruses. If a virus species cannot be estimated, then the extensive time and effort required to identify the virus can delay diagnosis and treatment or the determination of the cause of death. In such cases, exhaustive amplification using non-specific primers (the rapid determination system of viral genome sequences, RDV method) makes rapid virus identification possible [2]. Using cat fluid swab samples, we previously reported that the RDV method is useful for rapid virus identification in forensic samples from which it is difficult to estimate the virus species [3]. In recent years, next-generation sequencing (NGS) has been successfully used for virus identification in a clinical context [4,5] and for the detection of novel viruses [6,7].

In the forensic field, various materials, including body fluids, swabs, blood, and tissue, are handled. The methods for the collection and preservation of microbial forensic samples have been reviewed [8], and many methods for identifying viruses from samples have been described [9]. Several biocrimes have been reported [10,11,12], and the virus sequences obtained from the samples have been used for identification and molecular phylogenetic analysis. In this study, the influenza virus was inoculated into mice to prepare infected tissue samples as forensic samples. Assuming that the pathogen of this infected sample was unknown, the aim of this study was to rapidly identify the pathogen from the infected tissue samples. First, we attempted to quickly detect the causative agent using the RDV method. As viral genomic RNA was expected to be degraded in the tissue samples collected after death, the viral genes in the samples were quantified to examine whether detection using the RDV method was possible. Therefore, quantitative polymerase chain reaction (qPCR) procedures were developed. In addition to the RDV method, we attempted to detect the causative agent rapidly from the tissue samples using MiSeq and MinION NGS methods. MiSeq sequencing is performed using the reversible incorporation and subsequent detection of fluorescently labeled terminator nucleotides after DNA clusters are formed on the flow cell via bridge amplification [13,14,15]. For MinION sequencing, DNA is directed to a nanopore formed within a membrane by an attached motor protein. The changes in the baseline ionic current as the single-stranded DNA is translocated through the pore are recorded as the raw data, and the software processes the raw data from squiggles to the string of nucleotides [13,16,17]. MiSeq and MinION were also compared in terms of the mass of DNA required per sample and the library and sequence preparation time. In this study, we show that MinION may be useful for the rapid identification of pathogens from infected tissue samples.

## 2. Materials and Methods

### 2.1. Virus and Cells

Influenza A virus (H1N1) strain A/PR/8/34 was obtained from the American Type Culture Collection (ATCC, Manassas, VA, USA). Madin–Darby canine kidney (MDCK) cell line (RCB0995) was provided by the Riken BRC (Tsukuba, Japan) through the National BioResource Project of the MEXT/AMED and maintained at 37 °C under 5% CO_2_ in Eagle’s minimum essential medium (Sigma-Aldrich, St. Louis, MO, USA) supplemented with 10% fetal bovine serum (Thermo Fisher Scientific, Waltham, MA, USA) and 0.1 mM nonessential amino acids (Thermo Fisher Scientific) as instructed.

### 2.2. Virus Preparation and Titration

Virus preparation and titration were performed as described previously [18]. Briefly, viral stocks were obtained by inoculating MDCK cells with the influenza A virus. After the cytopathic effect was observed, the TCID_50_ value was calculated using the Behrens–Kärber method [19]. Viral stocks were stored at −80 °C until use.

### 2.3. Virus Inoculation

Five-week-old BALB/c female mice, which were used for virus inoculation, were obtained from Nippon Bio-Supp. Center (Tokyo, Japan) and were housed at room temperature (maintained at 23 ± 1 °C) with a relative humidity range of 50–64%. The mice were fed an MF rodent diet from Oriental Yeast Co., Ltd. (Tokyo, Japan) and allowed free access to water. Six mice were used for inoculation, and each mouse was intranasally inoculated with a 20-μL diluent (1.0 × 10^5^ TCID_50_) of the stock virus. After inoculation, mice were observed daily. Two mice died seven and nine days after inoculation, and each was dissected on that day (*n* = 2).

### 2.4. Sample Preparation

Homogenization was performed as described previously [20], with a few modifications. After dissection, each lung was weighed and placed in 0.4 mL of Eagle’s minimum essential medium (ATCC). Homogenization of lung tissue was performed using a pestle (AS ONE Corporation, Osaka, Japan) and microtube, and a 25% homogenate was prepared with Eagle’s minimum essential medium. Lung homogenates were centrifuged at 2000× *g* for 10 min at 4 °C, and supernatants were stored at −80 °C until use.

### 2.5. RNA Extraction and cDNA Synthesis

RNA was extracted using an AllPrep DNA/RNA Mini Kit (Qiagen, Hilden, Germany) from 50 μL of supernatant from the lung homogenates. cDNA was synthesized from 2 μL of the RNA extract using a random hexamer as the reverse transcription primer and the PrimeScript RT Reagent Kit (TaKaRa Bio, Shiga, Japan). For NGS, double-stranded cDNA was synthesized from 7 μL of the RNA extract using a random nonamer as the reverse transcription primer and a PrimeScript Double Strand cDNA Synthesis Kit (TaKaRa Bio). These procedures were performed in accordance with the manufacturer’s protocols.

### 2.6. Amplification and Quantification of the PB1 Gene

To examine whether detection using the RDV method was possible, the viral genes in the samples were quantified. Therefore, a qPCR procedure targeting the influenza A virus polymerase basic protein 1 (*PB1*) gene was developed. The primer sequences and amplicon lengths are listed in Table 1. Primer pairs to amplify the *PB1* gene were designed using Primer-BLAST (https://www.ncbi.nlm.nih.gov/tools/primer-blast/, accessed on 19 December 2015). Previously reported primers [21] were also used. The synthesized cDNA was amplified using TB Green Premix Ex Taq (TaKaRa Bio) and the LightCycler Nano (Roche Diagnostics, Mannheim, Germany). The following PCR conditions were used to amplify the *PB1* gene: an initial denaturation step of 30 s at 95 °C, followed by 40 cycles of denaturation at 95 °C for 10 s, and annealing and extension at 60 °C for 30 s. Next, a melting curve analysis was performed from 60 °C to 95 °C. A no-template control was used in each batch of PCR mixture as a negative control.

### 2.7. Measurement of the DNA Concentration

The DNA concentration of each PCR product was measured using the NanoDrop 2000 spectrophotometer (Thermo Fisher Scientific). For NGS, synthesized double-stranded cDNA was measured using the QuantiFluor ONE dsDNA System (Promega, Madison, WI, USA).

### 2.8. RDV Method

The RDV method was performed as described previously [3], with a few modifications. Assuming that the virus species was unknown, cDNA synthesized from lung homogenate RNA was used without a virus propagation step.

### 2.9. NGS

DNA libraries were prepared using the Nextera XT DNA Library Preparation Kit and the Nextera XT Index Kit (Illumina, San Diego, CA, USA) according to the manufacturer’s protocols. The libraries were normalized, pooled, and diluted for sequencing. Then, 24 μL of pooled library solution and 576 μL of hybridization buffer were mixed and sequenced using the MiSeq Reagent Kit v2 300 cycles (Illumina). For sequencing using MinION (Oxford Nanopore Technologies, Oxford, UK), DNA libraries were prepared using a Rapid Sequencing Kit (Oxford Nanopore Technologies) and sequenced with Flow Cell FLO-MIN106 R9 Version (Oxford Nanopore Technologies) in accordance with the manufacturer’s protocols. For taxonomic classification of reads generated by MinION sequencing, What’s in my Pot? (WIMP, rev. 3.2.1) in the EPI2ME workflow (Oxford Nanopore Technologies) was used. CLC Genomics Workbench 11 (Qiagen) was used to analyze all the obtained reads.

### 2.10. Statistical Analysis

Copy numbers were analyzed using paired two-tailed Student’s *t*-tests. A *p*-value < 0.05 was considered significant. Statistical analyses were performed using Microsoft Excel 2016 MSO (version 2111).

## 3. Results

### 3.1. qPCR Assay Development

**Figure 1:**
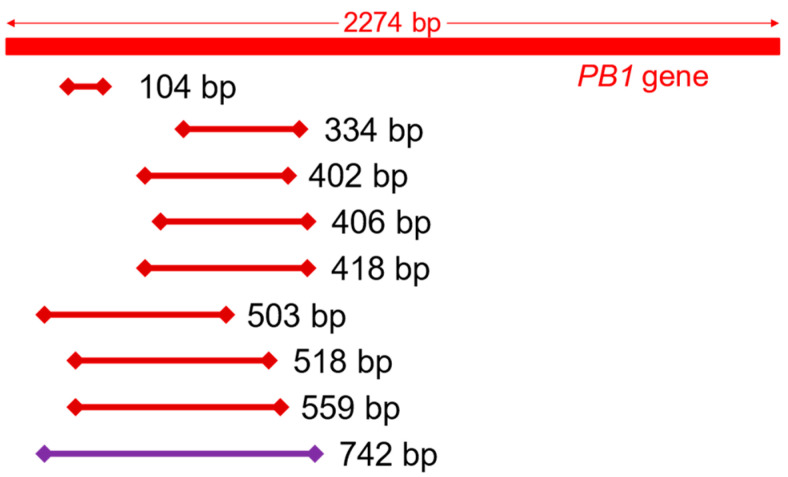
Positions of amplicons on the influenza A virus *PB1* gene. The 742 bp amplicon was used as a template for the standard curve.

The positions of nine fragments amplified by the primer pairs presented in Table 1 are shown in Figure 1. First, a 742-bp fragment was amplified using cDNA derived from the stock virus as a template. To generate a standard curve, the 742-bp PCR product was first purified. After the DNA mass of the purified product had been measured, the copy number was calculated. Using this product as a template, amplification using the eight primer pairs was conducted, and the presence or absence of non-specific reactions was examined. As a result, the primer pairs that generated amplicon lengths of 104-bp, 402-bp, and 559-bp were appropriate for quantification of the *PB1* gene. For each qPCR run, 2 × 10^3^ to 2 × 10^7^ copies of the 742-bp amplicon were included as standards. Fluorescence was monitored throughout the reaction, and the cycle quantification (Cq) value was determined. A standard curve was generated from the Cq value and the copy number. Quantitation was linear over the range of copy numbers examined (data not shown). The copy number of each sample was determined using the standard curve.

### 3.2. Quantitation of the PB1 Gene in Lung Homogenate Samples

**Figure 2:**
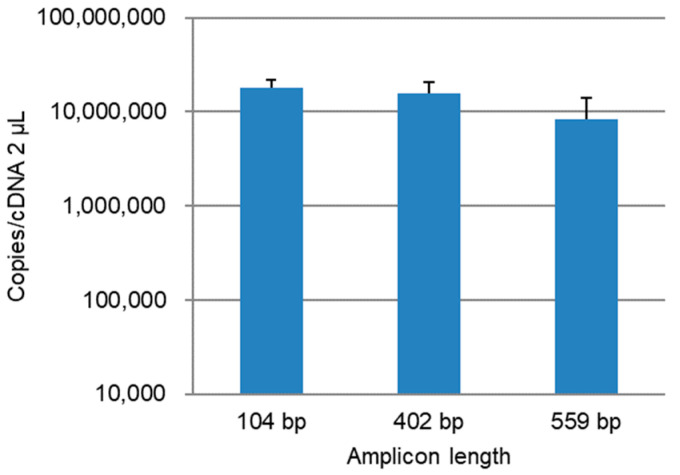
Quantitation of the influenza A virus *PB1* gene in the lung homogenate samples. Quantification was performed twice per sample, and the average of the two samples was calculated. Error bars indicate the standard deviations.

The copy number of the *PB1* gene in the lung homogenates was quantified by amplicon length. The copy number decreased as the amplicon length increased (Figure 2). The copy number at 559 bp was significantly lower than at 104 bp (*p* < 0.05) and 402 bp (*p* < 0.001) but was relatively high at 559 bp.

### 3.3. Detection with RDV

**Figure 3:**
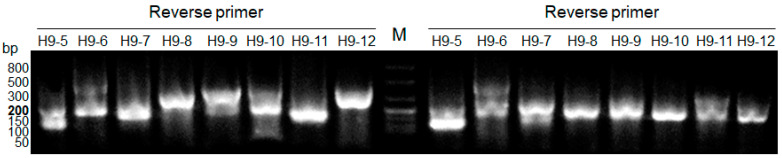
Partial electrophoretic results after amplification of the second library during the RDV method. In this amplification, H1–3 were used as forward primers, and H9–5 to H9–12 were used as reverse primers. Lane M, FlashGel™ DNA Marker, 50 bp–1.5 kb.

As a relatively high copy number was detected even for the 559-bp amplicon, the RDV method was performed using cDNA synthesized from the lung homogenate RNA. As shown in Figure 3, bands of various densities and lengths were obtained from the two cDNA samples. In total, 24 bands with lengths ranging from 100 to 500 bp were excised from agarose gels and purified. After direct sequencing, the obtained nucleotide sequences were searched using the Basic Local Alignment Search Tool (BLAST) program (https://blast.ncbi.nlm.nih.gov/Blast.cgi, accessed on 11 August 2021). The majority of reads (16 reads) were homologous to the 18S, 28S, and 45S ribosomal RNA genes of mammals, including mice (Table 2). Other reads were homologous to chromosomal sequences of mice and fish (four reads), mRNAs such as that of mouse tumor necrosis factor (three reads) and the 18S ribosomal RNA gene of nematodes (one read). However, no sequences were homologous to the influenza virus.

### 3.4. Detection with NGS

On average, approximately 1,800,000 raw reads were obtained from two double-stranded cDNA samples using MiSeq. To remove host reads, reads were mapped to the mouse genome after quality trimming. Then, 19 and 31 contigs were assembled from the un-mapped reads (55,019 and 82,712 reads, respectively). Of the 19 contigs, the minimum, maximum and average lengths were 202, 2191, and 768 bases, respectively. Of the 31 contigs, the minimum, maximum and average lengths were 225, 2535, and 673 bases, respectively. The 50 contigs were searched using the BLAST program. The majority of contigs (12 and 18, respectively) were homologous to influenza A virus sequences, although most others were homologous to mouse and bacterial ribosomal RNA genes (data not shown). In total, 45,997 and 142,475 reads were generated by MinION sequencing of the two samples, and these reads were analyzed with WIMP of the EPI2ME workflow. Of the 142,475 reads, 116,371 were host reads. Among the remaining reads, 155 matched influenza A virus sequences and these were detected most frequently. Of the 45,997 reads, 14,783 were host reads. Among the remaining reads, 32 influenza A virus reads were detected, following reads for *Escherichia coli*, Escherichia virus Lambda, *Pseudomonas fragi*, and *Escherichia albertii*. Although they accounted for fewer than 10 reads in both analyses, reads relating to endogenous viruses, including Escherichia virus, Enterobacteria phage, and *Mus musculus* mobilized endogenous polytropic provirus, were detected.

To confirm their identities, the obtained reads were mapped to the influenza A virus and mouse genomes (Table 3). The data obtained from the MiSeq reads of the two samples were mostly consistent, but the data obtained from the MinION reads varied. Although many un-mapped reads were detected among the MinION reads, almost the same number of reads mapped to influenza A virus as the WIMP analysis.

Finally, the MiSeq and MinION sequencing data were compared (Table 4). Although the library preparation kit for MinION sequencing requires 400 ng of input DNA per sample, the prepared mass was less than 100 ng. The time required for library and sequence preparation for MinION sequencing was approximately 17 h, which was much shorter than for MiSeq sequencing. The average read length of MinION sequencing was shorter than generally produced.

## 4. Discussion

When a pathogenic virus needs to be rapidly identified from an infectious forensic sample, the RDV method allows for detection of the virus sequence with equipment widely available in most laboratories [3]. In a previous report, norovirus genomes were detected from approximately 1 × 10^6^ copies of viral cDNA using the RDV method [22]. Although the copy number of cDNA used in our study was more than 1.7 × 10^6^, the target influenza virus genome could not be detected from the infected tissue samples. This copy number appeared to have been low to detect using the RDV method without the virus propagation step. Therefore, when the RDV method is used without the virus propagation step, it may require a larger number of samples; however, the number of samples that can be processed is limited. Although omitting the virus propagation step in the RDV method saves time and effort, this step may be necessary to reliably detect the target genome. As the host genome is often detected using the RDV method, the removal of the host genome is important. Although it is difficult to remove the host genome from samples in the RDV method, NGS allows the host genome to be removed during analysis.

Read analyses of MiSeq and MinION sequencing data revealed that the target virus species could be detected from the infected tissue samples. Although a huge number of reads can be obtained from a small mass of DNA using MiSeq, library and sequence preparation are time-consuming and labor-intensive. Although MinION requires a large mass of DNA, the library can be prepared in approximately 30 min per sample, and reads can be obtained in a shorter time. Furthermore, multiplex samples can be prepared together using the barcoding kit, and the analysis of the obtained reads can be started during sequencing. Although not performed in this study, these adaptations to the method can accelerate the process of pathogen detection. If the sample is a human organ, then a larger mass of DNA can be obtained, which may make MinION the appropriate detection method. Owing to its speed and convenience, MinION is also useful for human identification from skin microbiota and on-site sequencing [13]. In addition, MinION has been used for field-based forensic analysis [23]. The MinION platform is helpful in generating a large number of bacterial genomes [24,25].

Here, the number of reads produced by MinION sequencing was small and varied because the specified DNA mass was not available for library preparation. This may have had the effect of shortening the reads. The proportions of influenza A virus and mouse reads were lower with MinION than with MiSeq (Table 3), but this may reflect differences in accuracy. The accuracy of MinION sequencing was inferior to that of MiSeq in our study because the accuracy is lower [26]. However, the accuracy of MinION sequencing has improved over time [27], and this discrepancy between MiSeq and MinION is, therefore, expected to decrease. The improvements in MinION sequencing may lead to an increased number of mapped reads and a decreased number of unmapped reads.

This study confirmed that target virus species could be rapidly detected from infected tissue samples using WIMP. When a causative virus needs to be rapidly identified from an infectious sample, MinION may be an appropriate method. MinION can also be used to identify pathogens from tissue samples of infectious diseases of unknown cause. If the virus species is ascertained quickly, appropriate treatment can be initiated, or the cause of death can be resolved rapidly. Although PCR confirmation after species detection was not performed in this study, official identification requires PCR confirmation with species-specific primers.

We are currently investigating tissue samples harvested from influenza virus-infected mice a number of days after death. We will use MinION to determine whether the target virus can be detected from such samples.

## Figures and Tables

**Table 1:** Primers to amplify the influenza A virus polymerase basic protein 1 (*PB1*) gene.

Primer Name	Forward Primer Sequence (5′–3′) Reverse Primer Sequence (5′–3′)	Amplicon Length (bp)	Reference
- -	GATGGACAACAAACACCGAAACT TACACAATGTTTGGGCATAACC	104	[21]
PB1-334F PB1-334R	CAGATCAAATGGCCTCACGG ACTCCTTGCCAGTGTCTCAAC	334	This study
PB1-402F PB1-402R	GGAGGTTGTTCAGCAAACACG CCTGGGGTTGCAATTGCTCT	402	This study
PB1-406F PB1-406R	CCGACAGACCTATGACTGGAC CCTTGCCAGTGTCTCAACAA	406	This study
PB1-418F PB1-418R	GAGGTTGTTCAGCAAACACGA ACCCCCTTATTTGCATCCCTG	418	This study
PB1-503F PB1-503R	CTTACAGCCATGGGACAGGA AGTCATATTGTCTCTCACCCGTC	503	This study
- PB1-518R	GATGGACAACAAACACCGAAACT TGTGTTCAGGGTCAATGCTCT	518	[21] This study
- PB1-559R	GATGGACAACAAACACCGAAACT GCTCTCCGTTTTAGCTTCCC	559	[21] This study
PB1-742F PB1-742R	CCTCCTTACAGCCATGGGAC CTCCAACTGGCAACCCTGAT	742	This study

**Table 2:** Reads detected using the RDV method.

Read Detail	Number of Reads
18S ribosomal RNA genes of mammals	12
28S ribosomal RNA genes of mammals	2
45S ribosomal RNA genes of mammals	2
Chromosomal sequences of mice	2
Chromosomal sequences of fish	2
Mouse tumor necrosis factor mRNA	1
Mouse ribosomal protein S9 mRNA	1
Mouse Dab2ip mRNA	1
18S ribosomal RNA gene of nematodes	1
Total	24

**Table 3:** Mapping the obtained reads to the influenza A virus and mouse genomes.

	MiSeq Reads		MinION Reads	
	Sample 1	Sample 2	Sample 1	Sample 2
Mapped to influenza A virus	2142	2678	30	147
	(0.12%)	(0.15%)	(0.07%)	(0.10%)
Mapped to the mouse genome	1,800,025	1,659,092	10,829	77,556
	(97.03%)	(95.25%)	(23.54%)	(54.43%)
Un-mapped	52,877	80,034	35,138	64,772
	(2.85%)	(4.60%)	(76.39%)	(45.47%)
Total reads	1,855,044	1,741,804	45,997	142,475

**Table 4:** Comparison of the sequencing parameters between MiSeq and MinION in this study.

	MiSeq	MinION
Input DNA for library preparation	1 ng/sample	86 ng/sample
Time for library preparation	approximately 3 h ^1^	approximately 30 min
Time for sequence preparation	approximately 1.5 h	approximately 10 min
Total sequencing time	24 h	16 h
Average read length	132 bases	1603 bases

^1^ Six samples, including the control, were prepared simultaneously using the Nextera XT Index Kit (Illumina).
